# Protein network prediction and topological analysis in *Leishmania major *as a tool for drug target selection

**DOI:** 10.1186/1471-2105-11-484

**Published:** 2010-09-27

**Authors:** Andrés F Flórez, Daeui Park, Jong Bhak, Byoung-Chul Kim, Allan Kuchinsky, John H Morris, Jairo Espinosa, Carlos Muskus

**Affiliations:** 1 Programa de Estudio y Control de Enfermedades Tropicales-PECET, Universidad de Antioquia, Calle 62 No 52-59, Lab. 632, Medellín, Colombia; 2 Korean BioInformation Center (KOBIC), KRIBB, Daejeon, 305-806, Korea; 3 Agilent Technologies, Santa Clara, California, USA; 4 Department of Pharmaceutical Chemistry, University of California San Francisco, San Francisco, California, USA; 5 Grupo de Automática-GAUNAL, Universidad Nacional Sede Medellín, Medellín, Colombia

## Abstract

**Background:**

Leishmaniasis is a virulent parasitic infection that causes a worldwide disease burden. Most treatments have toxic side-effects and efficacy has decreased due to the emergence of resistant strains. The outlook is worsened by the absence of promising drug targets for this disease. We have taken a computational approach to the detection of new drug targets, which may become an effective strategy for the discovery of new drugs for this tropical disease.

**Results:**

We have predicted the protein interaction network of *Leishmania major *by using three validated methods: PSIMAP, PEIMAP, and iPfam. Combining the results from these methods, we calculated a high confidence network (confidence score > 0.70) with 1,366 nodes and 33,861 interactions. We were able to predict the biological process for 263 interacting proteins by doing enrichment analysis of the clusters detected. Analyzing the topology of the network with metrics such as connectivity and betweenness centrality, we detected 142 potential drug targets after homology filtering with the human proteome. Further experiments can be done to validate these targets.

**Conclusion:**

We have constructed the first protein interaction network of the *Leishmania major *parasite by using a computational approach. The topological analysis of the protein network enabled us to identify a set of candidate proteins that may be both (1) essential for parasite survival and (2) without human orthologs. These potential targets are promising for further experimental validation. This strategy, if validated, may augment established drug discovery methodologies, for this and possibly other tropical diseases, with a relatively low additional investment of time and resources.

## Background

Leishmaniasis is a complex infectious disease caused by several species of the *Leishmania *genus, affecting more than 2 million of people around the world in 88 countries. In addition to endemic countries, there have been increasing numbers of cases in non-endemic countries due to tourism [1-5]. The parasite is transmitted to human or animal reservoirs by the female insect of the genus *Lutzomyia *in the New World and *Phlebotomus *in the Old World [1]. Leishmaniasis has three main clinical presentations: cutaneous, mucocutaneous and visceral. The visceral form affects mainly children, who can die if adequate treatment is not provided in a timely manner. The cutaneous and mucocutaneous forms can cause severe disabilities in adults, affecting productivity in rural areas. At present, there are no available vaccines for this disease in spite of multiple research efforts [6]. The main measures for controlling the disease rely upon chemotherapy and vector control, which are tightly related given that human beings may act as reservoirs for the parasites in some endemic areas (antropozoonotic transmission). In spite of these measures, the number of cases continue to increase in many endemic countries, such as Colombia [7].

Current anti-leishmanial therapy has been unsuccessful due to toxicity, varying sensitivity of different *Leishmania *species, diversity of host immune responses, and different pharmacokinetics of the drug employed. The classical treatment for all forms of leishmaniasis has been pentavalent antimony in the form of sodium stibogluconate (Pentostam, Glaxo-Smith-Kline) or meglumine antimoniate (Glucantime, Rhone-Polenc). Severe side effects, including death, are associated with these compounds [8,9], and increasing resistance to antimonials is currently a major problem in many endemic countries [2,10]. Several drugs, such as Pentamidine and Amphotericin B, have also been used for leishmaniasis treatment. However, the presence of side effects, route of administration (injection rather than a pill), high cost, and differences in efficacy against the different clinical forms of the disease constrain their widespread use as drugs of choice. More recently, Miltefosine, an oral drug, originally developed as an antineoplastic compound, has been used successfully for treatment of visceral and cutaneous leishmaniasis [11,12], but with variable efficacy in Central and South America [13]. Moreover, a phase IV trial in India has shown an increment in the relapse rate with Miltefosine, indicating that drug resistance may develop quickly [14,15]. For all these reasons, there is an urgent need for new, safe, and cheap anti-leishmanial compounds.

Drug discovery efforts, through public private partnerships, for the primary protozoal parasitic diseases of the developing world --malaria, leishmaniasis, and trypanosomiasis -- have renewed the interest in developing new drugs and vaccines that can be accessible to the affected, primarily poor, population [16]. The drug discovery process begins with a search for drug targets that must fulfill two main requirements in the case of infectious diseases; (1) to be essential for the parasite survival and (2) to be specific, in that the target should not have a counterpart in the human host that can give rise to toxic effects. However, there is no consensus yet on the best biological indicators of essentiality. Indicators such as expression level and subcellular localization have been used to classify proteins as druggable. However, these assumptions often do not account for the complexity of the underlying biological network of interactions among those proteins [17].

New research initiatives have been undertaken to collect genome sequences along with high-throughput expression and proteomic data from different organisms. This constitutes an important source of biological information that can be employed efficiently in the search for new drugs for a large number of human and veterinary diseases. Bioinformatics tools have enabled researchers to extract and manipulate this biological information with the goal of understanding protein function. Unfortunately, the knowledge of the functions of proteins in their native form has not yet provided us with an understanding of the complexity of cellular behavior, thus there is not yet a clear definition of essentiality. Proteins inside the cell typically do not function in their native state alone, but rather by interacting in concert with other proteins, generating a high dimensional network with a complicated structure. Because of the networked nature of protein function, topological analysis of the protein network may help to identify essential proteins that can be potentially drug or vaccine targets. Recent studies carried out with experimental protein interaction networks of *Saccharomyces cerevissiae *and *Caenorhabditis elegans*, [18,19] have confirmed the effectiveness of topological metrics in predicting protein essentiality, demonstrating strong correlation with knockout and knockdown data. These studies have also expanded to organisms of medical importance, such as the protozoan parasite *Plasmodium falciparum *[20], in the interest of discovering new drug and vaccine targets. This data is available through the system PlasmoID [21]. Topological analysis has also been useful in detecting important proteins, even when the protein network has been predicted using an orthology-based method, as in the case of the human interactome[22].

In this work, we predicted the protein network of *Leishmania major *using protein sequences via three methods, iPfam, PSIMAP and PEIMAP. We analyzed the predicted protein network with the metrics of connectivity and betweenness centrality, in order to identify essential proteins. Protein interaction data were analyzed to detect GO enriched clusters, to determine the possible pathways of detected targets, and to infer the biological processes performed by proteins with unknown functional description. The list of putative protein targets is a starting point for experimental validation by *in vitro *assays and further discovery of new anti-leishmanial drugs.

## Methods

### Protein network prediction using PSIMAP, iPfam and PEIMAP

Predictions of protein-protein interactions (PPIs) were generated using the pipeline previously designed and applied in *Xanthomonas oryzae *[23], employing three different methods: PSIMAP, iPfam, and PEIMAP.

### PSIMAP

PSIMAP http://psimap.com/[24] infers interactions between proteins by using interacting domain pairs from known PDB (Protein Data Bank) structures. We extracted protein sequences of *Leishmania major *from the GeneDB database ftp://ftp.sanger.ac.uk/pub/databases/L.major_sequences/DATASETS/LmjFwholegenome_20070731_V5.2.pep. We aligned these sequences using PSI-BLAST [25] against the SCOP 1.71 database[26] with an E-value cutoff of 0.0001, as described previously in [23]. We predicted a total of 158,984 interactions for 3,184 proteins by applying PSIMAP [27] domain pairs to the domain assignment. The original definition of interaction in this database is based on atomic distance between domains in the structures of protein complexes.

### iPfam

We analyzed iPfam interactions using domain assignments from Pfam release 18.0 [28] using the tool hmmpfam with an E-value cutoff of 0.01. By integrating them with Pfam domain interaction pairs from iPfam [29], a total of 50,398 predicted protein-protein interactions were constructed from 2,336 *Leishmania *proteins.

### PEIMAP

We aligned *Leishmania *proteins with the PEIMAP database http://peimap.kobic.re.kr using BLASTP [25] with a minimal cutoff of 40% sequence identity and 70% length coverage. The PEIMAP database includes protein-protein interaction (PPI) information from six source databases: DIP, [30] BIND, [31] IntAct, [32] MINT, [33] HPRD, [34] and BioGrid [35]. A total of 14,839 interactions were extracted involving 718 *Leishmania *proteins.

### Selecting confident predicted protein interactions

We used the 'combined score' method, applied in [23] and also used in the STRING database [36]. This method takes into account the reliability of each method (PEIMAP, PSIMAP and iPfam), assuming independence among them. The score is calculated according to the formula:

$$score=1-\prod_{i\;\;\in E}{\left(1-R_{i}\right)}^{n}$$

Where *score *is the confidence score, *E *is the set of methods under analysis (PEIMAP, PSIMAP, iPfam); *R_i _*is the reliability of method *i, n *is the number of interactions predicted by method *i*. The reliability score of PEIMAP comes from previous reported data [37] that takes into account the reliability of each experimental method for detecting protein interactions. The reliability score of iPfam is extracted from the score between two Pfam domains from iPfam database. Finally, the reliability score of PSIMAP uses the calculated distance between interacting structural domains (SCOP).

The final score was further normalized to the range of 0.0 to 1.0 combining all the scores. We selected 1,366 *Leishmania *proteins participating in 33,861 high-confidence PPIs, (confidence score >0.7), combining the results from the three methods employed (Additional file 1: Cytoscape network of Leishmania interactome). To evaluate the confidence of the metric results, the clustering coefficient and mean shortest path were compared against 1,000 random networks generated with the Random Network Plugin in Cytoscape [38], and empirical p-values were computed.

### Detection of essential proteins with topological metrics and homology filtering with human proteome

Power law fit for the protein network was calculated using Network Analyzer v.2.6.1 [39]. Network topology metrics, such as betweenness centrality, connectivity, and the Double Scoring Scheme (DSS) were used to detect essential genes, using the Hubba server http://hub.iis.sinica.edu.tw/Hubba. This method takes into account weighted edges (confidence scores). The calculations were done over the largest component of the network, with 0.7 confidence cutoff. This cutoff was chosen to better fit the data with a power law distribution of the network. The detected targets were filtered by discarding *Leishmania *orthologs to human proteins.

### Clustering and GO enrichment analysis

We conducted cluster analysis of the largest component in the network in order to detect protein complexes and pathways. We used the Markov Clustering (MCL) algorithm [40,41], which has been demonstrated as a robust and fast algorithm to detect clusters in protein networks [42], using the implementation in the NeAT tool [43]. For proteins of unknown function in the GeneDB database http://www.genedb.org/Homepage/Lmajor, we predicted their possible biological roles by evaluating the results of GO enrichment analysis, using the BinGO plugin for Cytoscape.

## Results and Discussion

We constructed a protein-protein interaction (PPI) map, combining the results generated by PEIMAP, iPfam and PSIMAP. Despite the absence of protein interaction data for *Leishmania major *and the fact that protein interaction data from single organisms may contain some false positives that can bias the results, the use of interaction data from different species can help to reduce the noise in the predicted network [44]. Comparison to random networks and utilization of experimental evidence that confirms the essentiality of some of the predicted targets are indirect ways of validating the calculated PPI map. Other studies have successfully applied this approach to discovering drug targets using computational methods to predict protein networks, e.g. blast rice fungus, *M. tuberculosis*, and *Homo sapiens *[45-47]. The predicted *Leishmania major *interactome can be a starting point for future experimental PPI maps in *Leishmania*, particularly given the fact that many interactions may require post-translational modifications that may not occur in yeast [48], thus making it difficult to perform yeast-two-hybrid assays in this organism. The entire predicted network comprises 3,991 nodes and 190,708 interactions (including self loops and duplicate edges). The reduced coverage is likely due to the inability to perform domain assignment to several proteins in *Leishmania*. Only 18.0% of the *Leishmania *proteome is conserved across species (as defined in the CluSTr database: http://www.ebi.ac.uk/integr8/ClustrAnalysisPageOnly.do?orgProteomeID = 21780). This is a common limitation of orthology-based methods for protein network prediction.

It has been proposed that biological networks follow a power law distribution that corresponds to scale-free topology [49]. This is a global property of biological networks and it is important for a reliable prediction of essentiality when the metrics of connectivity and betweenness centrality are used. We performed the fitting of the node degree distribution to a power law using the least squares method, to determine if our predicted network was consistent with scale free topology. This resulted in an exponent of -0.867 (R^2^= 0.556) for the 0.60 confidence network. However, the calculated distribution for the 0.70 of confidence network showed an appreciable increase of the R^2 ^coefficient to 0.758 and the exponent to -1.199. This result does not correlate well with power law distribution, possibly because subnetworks can have a different degree of distribution compared to the entire interactome. Moreover, it has been pointed out that geometric models could fit better than power law distribution [50,51]. In spite of these limitations, we chose the 0.70 confidence cutoff, given that the network generated by applying this cuttoff fits better with a scale-free topology. This also enabled us to claim with more confidence that a detected hub and bottleneck node may be essential for the network.

### Indentifying putative drug targets

Once the power law distribution is partially confirmed, other topological characteristics can be biologically meaningful. With this in mind, we conducted local topology analysis to identify hubs and bottlenecks that could be putative drug targets. We calculated connectivity and betweenness centrality over the 1,366-node network with 33,861 interactions (> 0.70 confidence). For all of these calculations we used the largest component and excluded isolated components from the larger original network, mainly because betweenness centrality, which calculates the number of shortest paths through a particular node, may generate an infinite number of shortest paths from isolated nodes, which can become confusing and make interpretation more difficult. The clustering coefficient and the mean shortest path of the network were compared against 1,000 random networks, (Table 1). We found that our protein network is more highly connected when its clustering coefficient is compared against the clustering coefficient values of the randomly generated networks. These results suggest that our network exhibits a modular architecture like other biological networks. This makes us more confident that the clusters might correlate with biological pathways. The mean shortest path is also significantly different from that of the random networks.

**Table 1:** Comparison of topology metrics of the predicted network versus random networks.

*Metric*	*Predicted Network*	*Random Networks*	*Empirical P-value*
Clustering Coefficient Average	0.70	0.18 ± 0.003	< 0.001

Mean Shortest Path Average	3.91	2.42 ± 0.003	< 0.001

The predicted network shows significant differences when compared to 1000 generated random networks preserving the node degree.

It has been shown that measures of connectivity [52] and betweenness centrality [53] improve the identification of essential proteins in protein networks [54]. Betweenness centrality correlates more closely with essentiality than connectivity, exposing critical nodes that usually belong to the group of scaffold proteins or proteins involved in crosstalk between signalling pathways (called *bottlenecks*). This metric has also been proposed in the new paradigm of network pharmacology as a good feature for investigating potential drug targets [55]. In the *Leishmania *major network, we selected the top 10% of the connectivity ranking as *hub *nodes and 20% of the betweenness centrality ranking for *bottlenecks*, according to previous methods for selecting such cutoffs [54,56]. In addition, a recently developed tool, HUBBA [57], provides an alternative way of prediction of essential nodes by the combination of two metrics: DMNC (Density of Maximum Neighborhood Component) and MNC (Maximum Neighborhood Component). Together, they are referred to as the Double Scoring Scheme (DSS). We applied the DSS system to our high confidence network with the goal of extending the range of potential drug targets. We chose the cutoff of the top 10 proteins identified by this tool, because that cutoff identifies the group with the highest probability to be essential (close to 100%). However, we found that this group overlaps with the group of detected hubs.

**Figure 1:**
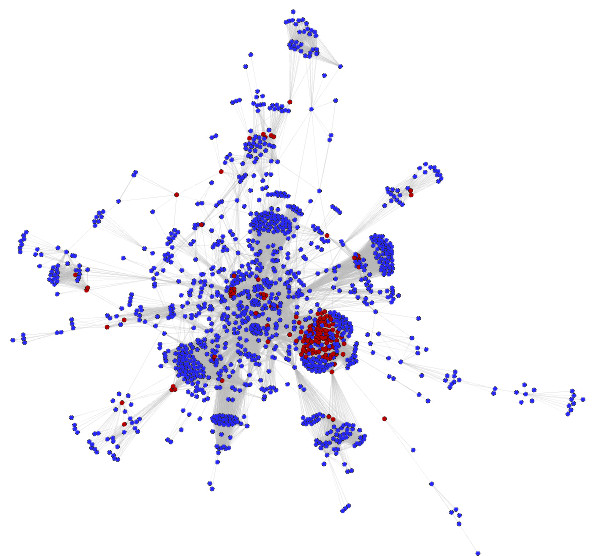
**Cytoscape network for the *Leishmania major *interactome**. The nodes highlighted in red are predicted essential nodes without human orthologs

In this first detection, which combines the results from connectivity, betweenness centrality, and DSS, we identified 384 potential targets, shown in Additional file 2: table S1. Once detected, targets need to be checked for orthologs in the human proteome, given that some drugs that bind conserved sites would perturb the corresponding human protein with possible toxic consequences. Utilizing the list of *Leishmania *orthologs to human proteins from the TDR database, we filtered the list of targets, removing those with homology to human proteins. The ortholog detection in the TDR database was performed using the OrthoMCL algorithm, which has shown high sensitivity compared to other methods[58], feature that it is critical to identify all of the possible human orthologs of *Leishmania *proteins. Once the *Leishmania*-human ortholog proteins were ruled out, the total number of potential proteins targets was reduced to 142 (Additional file 3: table S2). The network visualization of the targets is shown in Figure 1.

### Gene Ontology enrichment analysis and function prediction

It has been shown that detecting modular structures inside a biological network provides insights into the functional organization of cellular processes in living organisms [59]. In addition, it has been recognized that cluster detection combined with functional enrichment analysis enables the prediction of the biological function of proteins associated with a cluster [60]. We applied the MCL algorithm to generate clusters in the network, setting an inflation value of 1.8 and considering edge weights (confidence scores) for the calculations. Functional enrichment was carried out using BinGO, importing the *Leishmania major *annotation from Gene Ontology (GO) http://geneontology.org. We generated 63 clusters for the network. For each of those clusters, we assigned the most significant GO biological process. These results are shown in Additional file 4: table S3.

Close neighbours in protein interaction networks are frequently involved in similar processes and it has been shown that 70-80% of proteins in a cluster share at least one function. This implies that any unclassified protein could be tentatively assigned the function of its neighbours [60,61]. We found that 263 proteins without functional description in the GeneDB database are related to well-defined clusters. We assigned a biological process to those proteins based on the probability of membership in a specific GO enriched cluster. By this method, we predicted new protein roles for *Leishmania major *that were previously unknown using current annotation procedures (Additional file 5: table S4).

**Figure 2:**
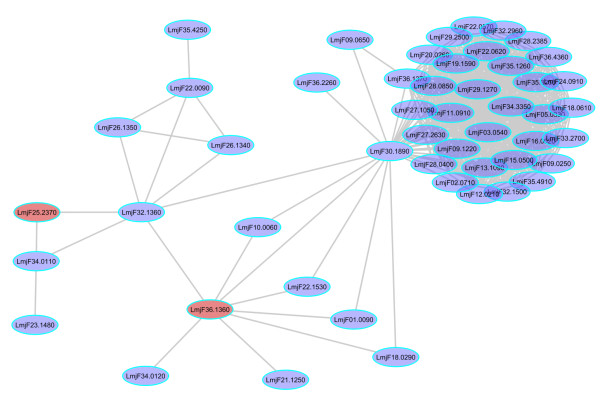
**Correspondence between topological analysis and flux balance analysis.** Partial representation of the Leishmania major interactome. Red nodes represent the double knockout predictions from the Leishmania metabolic network. The node LmjF36.1360 was present in the double knockout pair and also predicted as essential in the protein network. Betweenness centrality enables checking for redundancy.

The largest cluster contains 15% of the proteins in the network. They participate mainly in protein amino acid phosphorylation (GO:0006468) (p-value < 0.00001). Within the group of detected targets with no human counterpart, we found that 64% of the targets were also enriched in the protein amino acid phosphorylation process (Figure 2, Additional file 6: table S5), followed by proteins involved in nucleosome assembly (GO:0006334) 8%, nucleic acid metabolic process (GO:0006139) 4%, electron transport (GO:0006118) 4%, transport (GO:0006810) 4%, and protein amino acid alkylation (GO:0006139) 2%. The remaining proteins were distributed across processes with one protein per process and classified as 'other'; these accounted for 14% of the target proteins. This analysis suggests phosphoproteins as the main group to characterize and explore as drug targets. Proteins involved in nucleic acid metabolism also should be explored as possible drug targets, given that *Leishmania *does not have the enzymatic machinery to synthesize purines *de novo *[62]. Interestingly, proteins associated with nucleosome assembly appear as alternative options.

### Experimental evidence of the essentiality of predicted targets

As mentioned above, there is a significant proportion of phosphoproteins in the group of essential genes. This is plausible, given that these proteins are important regulators of differentiation and cell proliferation in many eukaryotes. However, it has been pointed out that the *Leishmania *kinome has particular distinctions from other eukaryotic kinomes (for a good review see reference [63]). We identified 91 kinases that were predicted as essential proteins in the network with no homology to the human kinome. This is an interesting and new group of potential targets for future drug screening in this organism, perhaps by using transfectant parasites as in the methodology developed by our group [64]. Within this group of kinases, LMPK [GeneDB:LmjF36.6470] has been experimentally shown as essential in *Leishmania mexicana*[65] with orthologs in *L. amazonensis*, *L. major*, *L. tropica*, *L. aethiopica*, *L. donovani, L. infantum*, and *L. braziliensis*[66]. There is a growing interest in this protein as a drug and vaccine candidate, given its importance in parasite proliferation at the amastigote stage.

**Figure 3:**
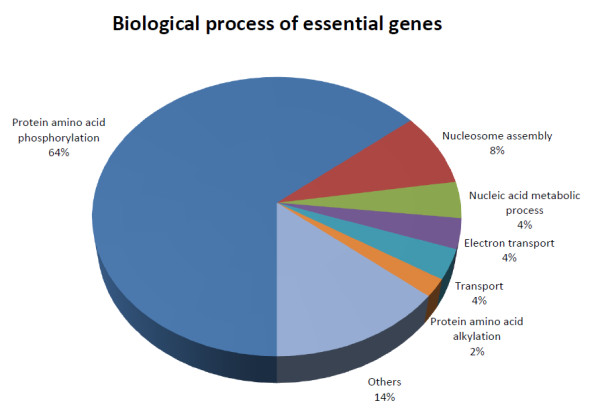
**Classification of essential genes according to GO biological process**. Phosphoproteins are overrepresented in the group of essential genes. (See text for  details).

A previous study has reconstructed the metabolic network of *Leishmania major *from literature and carried out flux balance analysis to predict potential drug targets [62]. However, when we compared the list of single predicted knockouts found by modelling with the list targets derived using topological methods, we did not find any overlap. This could be due to the fact that metabolic networks connect proteins by the metabolites that they catalyze and not by direct interaction. However, when we analyzed the double knockouts list, we found that the protein [GeneDB:LmjF36.1360] adenylate kinase, was predicted to be essential in our network and was also present in the double knockout pair of the metabolic network [GeneDB:LmjF36.1360,LmjF25.2370]. This is highlighted in red on Figure 3. This implies that redundancy in metabolic networks can also be detected by computing betweenness centrality in protein networks. Inhibition of this protein caused low growth in *L. donovani *promastigotes [67] and homology searching identified orthologs in *L.braziliensis, L.infantum, T. brucei, and T. cruzi*,. This would be advantageous in developing a drug for a wide spectrum of tropical diseases. Additionally, a DrugBank http://www.drugbank.ca search showed that the drug Gemcitabine could also have an inhibitory effect upon this protein, illustrating the potential use of this drug for tropical diseases besides its current use in cancer.

[GeneDB:LmjF35.1180 and GeneDB:LmjF35.0830] are fumarate reductase and fumarate reductase-like proteins that have been predicted as essential in our network. Neither has a human ortholog. Some reports have shown that compounds such as chalcones [68] and aurones [69] have a very potent inhibitory effect on these enzymes, making them interesting compounds for future drug development.

Three ABC transporters that were *Leishmania *specific - [GeneDB:LmjF34.0670, GeneDB:LmjF27.0470, GeneDB:LmjF32.2060] - were also predicted as essential. They confer resistance to antimonials and pentamidine by extruding the drug outside of the cell. Some research groups are investigating inhibitors for this family of transporters [70], with the goal of reverting the resistant phenotype to a susceptible phenotype. Based upon our analysis, we also identify these proteins as putative drug targets because of their essential role in the homeostasis of the parasite intracellular environment.

A final example that corroborates our findings with experimental data is the detection of sterol 24-c-methyltransferases [GeneDB:LmjF36.2390, GeneDB:LmjF36.2380] as essential and exclusive in our network. Those enzymes are involved in biosynthesis of ergosterol, which is a target pathway in *Leishmania *and fungi given its exclusivity and essentiality. Also, a recent study identified methyl-transferase as a promising drug target in *Cryptococcus neoformans *[71]. Moreover, this enzyme has been recently tested as an effective vaccine candidate in visceral leishmaniasis [72].

Finally, we looked at the expression level of exclusive predicted targets in the microarray data reported by Leifso et al.,[73], and we did not find any significant overexpression of the predicted essential genes at the amastigote stage. This could be expected, given that few genes have been found to be up or down-regulated across promastigote and amastigote stages. This suggests that essentiality could not be related to gene expression in the case of *Leishmania*, given regulation of protein abundance probably occurs at post-transcriptional level[73].

## Conclusion

This work constitutes the first attempt to explore protein interaction networks in the *Leishmania major *parasite by utilizing *in silico *methods. We have provided a putative list of essential proteins; some of them backed experimental evidence reported in literature. Of special interest are the predicted essential kinases that constitute an important group of Leishmania proteins to be explored as sources of new drug targets, given that they are important for parasite survival while having no homology to the human kinome. Further experimental studies are required to identify specific inhibitors. These results will aid future drug discovery efforts for this disease, enabling drug development in a more timely and cost-effective manner.

## Authors' contributions

AFF generated the idea, carried out topology and clustering analysis and wrote the manuscript. DP, JB. BCK helped with predicting the interactome and calculating confidence scores. AK helped with the topology and clustering analysis, JHM and JE reviewed it critically. CM supervised the project, provided biological information about *Leishmania *and wrote the manuscript. All the authors have read and approved the final manuscript.

## Supplementary Material

Additional file 1 **Cytoscape network of Leishmania interactome**. *Leishmania major *interactome in Cytoscape format with the annotation and topological metrics as Cytoscape attributes. Click here for file

Additional file 2 Table S1: List of targets detected by connectivity and betweenness centrality but not filtered for human homology. Click here for file

Additional file 3 Table S2: Final list of targets, excluding those with human orthologs from table S1. Click here for file

Additional file 4 Table S3: Clusters IDs from the whole network with overrepresented GO codes and p-values. Click here for file

Additional file 5 Table S4: List of hypothetical proteins with predicted biological process derived from the clustering and enrichment analysis. Click here for file

Additional file 6 Table S5: Number of essential genes for each GO Biological process. Click here for file
